# Delayed Neuropsychiatric Sequel Following Pediatric Carbon Monoxide Poisoning: A Case Report and Literature Review

**DOI:** 10.3389/fped.2022.861254

**Published:** 2022-05-02

**Authors:** Hila Gavrieli, Iris Noyman, Eli Hershkovitz, Benjamin Taragin, Guy Hazan

**Affiliations:** ^1^Department of Pediatrics, Soroka University Medical Center, Beersheba, Israel; ^2^Pediatric Neurology Unit, Soroka University Medical Center, Beersheba, Israel; ^3^Department of Radiology, Soroka University Medical Center, Beersheba, Israel

**Keywords:** carbon monoxide poisoning, delayed neuropsychiatric sequel, encephalopathy, delayed neurological sequel, seizure, neurological disorders

## Abstract

Carbon monoxide (CO) poisoning is a serious health problem. The main pathophysiological mechanism of acute CO poisoning is hypoxia due to the formation of carboxyhemoglobin (COHb). Delayed neuropsychiatric sequel (DNPS) occurs following an interval of several days to several weeks post-CO exposure and can present in many different manifestations, ranging from behavioral and mood disorders to encephalopathy and seizures and cause long-term neuropsychiatric sequel. The pathogenesis of DNPS following CO poisoning is a complex one that encompasses hypoxia-induced encephalopathy as well as inflammation, direct cellular changes and damage. The incidence varies and treatment is debated. We display a case of a previously healthy 13-year-old boy suffering from DNPS, presenting with seizures and encephalopathy and later developing optic nerve damage. Increased awareness to this condition might help diagnose future patients and aid in the understanding of the pathogenesis and treatment options for this poorly understood condition.

## Introduction

Carbon monoxide (CO) poisoning is a serious health problem resulting in approximately 50,000 annual visits to emergency departments in the United States (1–3). CO is a colorless, odorless, tasteless, and non-irritating gas and a common lethal toxicant due to an accidental or intentional (suicidal) exposure. The outcome of exposure ranges from subtle cardiovascular and respiratory effects to neuropsychiatric and other systemic complications and even fatality (1–3).

Delayed neuropsychiatric sequel (DNPS), also known as delayed encephalopathy (DE) or delayed neurological sequel (DNS), occurs following interval of days to several weeks post-exposure and after the disappearance of the symptoms of acute CO poisoning. The risk factors, pathogenesis, post exposure prevention, and efficient treatment are complex (1, 2, 4, 5).

The aim of this manuscript is to present a case of DNPS in a child intoxicated by CO.

## Case Presentation

A previously healthy 13-year-old boy was hospitalized following CO poisoning after breathing in a gas-heated shower. His younger brother was found dead at the scene. The period they were exposed to CO is unknown. The child was brought to the hospital intubated and sedated. After stopping sedation, his Glasgow Coma Scale was 7. Physical examination was normal. The patient’s carboxyhemoglobin (COHb) at arterial blood gases was of 23.6% (normal range < 3% in non-smokers). He had moderate respiratory acidosis; pH – 7.17, PCO2 – 75 mmHg, HCO3 – 27.4 mEq/L. The Troponin level was 58 ng/L and later increased later increased to 139 ng/L (normal range, 0.00–14 ng/L). On echocardiogram examination, a decrease in cardiac contractility was noted with ejection fraction (EF) of 45% (normal ≥ 50%).

**FIGURE 1:**
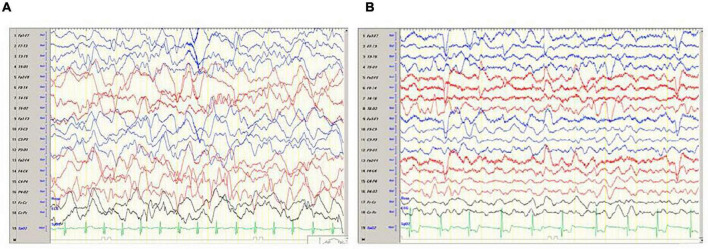
**(A)** Electroencephalogram (EEG) done on day 6 following the CO poisoning and a day after the onset of seizures. The EEG demonstrates severe encephalopathy with no focal findings. **(B)** EEG done on day 10 following the CO poisoning and 4 days after the onset of seizures. EEG demonstrates improvement background activity of Theta waves with several Delta waves.

**FIGURE 2:**
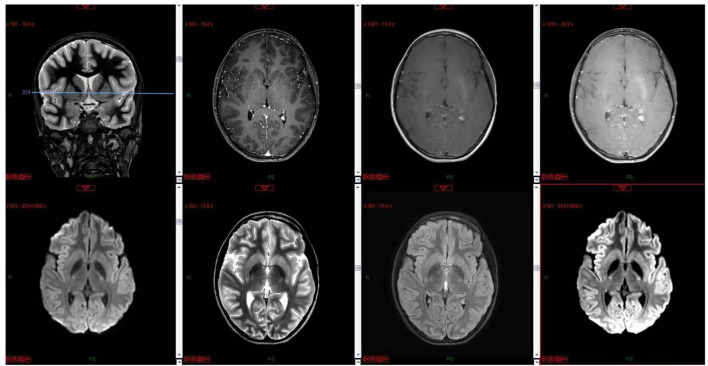
MRI done on day 9 following the CO poisoning and 3 days after the onset of seizures. The MRI of the basal ganglia in a single coronal localizer image and multiple axial images including T1 pre- and post-contrast, diffusion, FLAIR, T2 at the level of the basal ganglia show normal signal within the basal ganglia and deep nuclei.

The child was transferred to a high-pressure oxygen chamber where he received three consecutive treatments of about 2 h each within 24 h – the first two treatments were at 2.4 atmospheres and the 3rd treatment at two atmospheres. By the end of the hyperbaric treatment, he returned to full and normal apparent base-line neurological function. His COHb decreased to 1.1%, while echocardiogram examination demonstrated normal heart function. He was discharged home without any apparent neurologic impairment 5 days after the CO poisoning but returned on the same day complaining of weakness and headache. His neurologic examination revealed no pathological findings. Later that day, he had multiple seizure episodes; some were focal seizures, involving mainly the left arm accompanied with mouth automatism; others were generalized tonic clonic, requiring treatment with midazolam and phenytoin. Several hours later, the patient gained consciousness and became aware of his surroundings, but his neurological examination was abnormal and consistent with encephalopathy. Myoclonic jerks and repetitive dystonic/choreic movements of the limbs were noted. Muscle tone was increased in all limbs; hyperactive tendon reflexes with cross adduction and bilateral positive (extensor) Babinski reflex were found. Non-contrast and contrast enhanced, including the venous phase, computed tomography (CT) was within normal limits; electroencephalogram (EEG) demonstrated diffuse slow delta waves, consistent with encephalopathy without focal findings or epileptiform activity (Figure 1A). On the 8th day, following the CO poisoning, there was improvement in involuntary movements and marked improvement of muscle tone. Brain magnetic resonance imaging (MRI) demonstrated no pathological findings (Figure 2). The encephalopathy later improved, and involuntary movements decreased further. Repeated EEG demonstrated improvement of background activity (Figure 1B), consistent with the clinical improvement, and the child was discharged from the hospital.

The patient complained of vision impairment 19 days following the CO poisoning. Neuro-ophthalmologic examination revealed visual acuity of 6/10 in both eyes and impaired color vision, while visual fields by confrontation showed binocular peripheral vision loss. Fundus examination showed mild disk elevation in both eyes with the left eye nasal border slightly blurred (non-hyperemic) and mild temporal pallor in the right optic disk.

Two months after CO poisoning, the patient reported occasional myoclonic jerks of the right arm, some memory difficulties, and improvement of vision. Neurological examination disclosed clumsy alternating rapid movements. Bilateral sectorial paleness in the temporal regions of the optic disks was noted. EEG demonstrated further improvement of the background activity without interictal epileptiform discharges.

Seven months after the CO intoxication, flesh visual-evoked potentials (FVEP) test demonstrated a defect in nerve conduction and brain response to light in both eyes.

## Discussion

Delayed neuropsychiatric sequel can present in many different manifestations, including memory loss, behavioral and mood disorders, subtle cognitive deficits to severe dementia, vision loss, psychosis, and Parkinsonism (1–6). Incidence is estimated to be anywhere from 3 to 67% (1–4, 7–9).

The pathogenesis of CO poisoning includes tissue hypoxia caused by decreased oxygen delivery due to the formation of COHb, which has 200 times greater affinity to hemoglobin than oxygen (1–3) and direct cellular changes demonstrated in experimental models (10–12). These changes involve nitric oxide (NO) generation, mitochondrial oxidative stress, and inflammation mediated by several mechanisms, such as astrocytes-derived microparticles, platelets, and neutrophils activation (1, 2, 10–12). CO poisoning also causes adduct formation between myelin basic protein (MBP) and malonylaldehyde, a reactive product of lipid peroxidation, resulting in an immunological cascade, contributing to the development of delayed CO neuropathology (13).

A study that compared the pathophysiological features of DNPS vs. hypoxia alone (in randomized groups of rats) demonstrated distinct differences between the groups: (1) demyelination induced by CO is more severe and lasts longer than that induced by hypoxia alone; (2) microglial cells’ number in the hippocampus was decreased after CO exposure; and (3) neurotrophic factors are decreased in the hippocampus after CO exposure as compared with exposure to low oxygen (4).

In patients suffering from DNPS, focal and generalized neuroanatomical abnormalities are observed on MRI and CT imaging. Acute demyelination, damage to the sub-cortical white matter, and cerebral atrophy are seen. Focal structural lesions are reported in the thalamus, basal ganglia, hippocampus, corpus callosum, white matter, and fornix (14).

A patient’s initial presentation following CO poisoning does not predict the development of DNPS, but some factors have been shown to increase the risk of its development, such as older age, long exposure to CO, and myocardial injury (7–9). Elevated inflammatory markers have also been proposed to increase the risk for DNPS (7, 9).

Hyperbaric oxygen (HBO) therapy has been suggested both as preventive measure and as treatment for DNPS- for the short term by eliminating CO and raising oxygen partial pressure and for the long-term sequel by reducing oxidative stress and inflammation (1, 2, 10, 12, 15).

A large double-blind randomized control study on 152 patients with acute CO poisoning (less than 24 h from exposure), including patients with severe poisoning leading to loss of conscious, demonstrated that HBO therapy reduced cognitive sequelae when assessed after 6 weeks and after 12 months from insult compared to therapy with 100% O2 administration (16).

The prophylactic effect of HBO therapy on DNS was related to the inhibition of MBP-induced lymphocyte activation after CO poisoning (10).

However, it was found in two randomized controlled trials that, in patients with transient loss of consciousness, there was no evidence of superiority in terms of “complete” neurologic recovery after 4 weeks of HBO over normobaric oxygen therapy (17). In comatose patients, two HBO sessions were associated with significant worse outcomes than one HBO session (17).

In a recent review on the role of HBO in CO poisoning, its biologic effects were emphasized: HBO inhibits lipid peroxidation transiently, reduces leukocyte adhesion to injured microvasculature, and reduces brain inflammation caused by the CO-induced adduct formation of myelin basic protein. It also improves mitochondrial function (18). Therefore, the author suggested considering HBO therapy for all cases of acute symptomatic CO poisoning (18).

The effects of dexamethasone combined with HBO have been compared in a randomized clinical trial in 120 patients with DNPS after acute CO poisoning HBO monotherapy (19). After 4 weeks of treatment, the experimental group had a significantly higher average mini-mental state examination (MMSE) score and a significantly lower average National Institutes of Health Stroke Scale (NIHSS) score compared to the control group (19). The level of MBP in the CSF of patients was significantly lower in the experimental group than in the control group (19). The long-term effect of combination therapy of 3-N-butylphthalide, a Chinese medication for cerebrovascular disease, dexamethasone, and HBO, was compared to patients with DNPS receiving HBO monotherapy (20). At 1 month, 3 months, and 1 year after the treatment, cognitive scores were all significantly higher in the combined therapy group than those in the control group (20).

To conclude, DNPS can range from mild mood and behavioral changes to encephalopathy and seizures (as seen with our patient). Due to its unpredicted nature (of CO poisoning and the development of the neuropsychological sequel) and lack of routine baseline neuropsychiatric evaluation in most patients, it is extremely difficult to assess the true incidence and consequences of DNPS. In a literature review, there are very few case reports and even fewer pediatric case reports of DNPS (3, 5, 21, 22). It seems that DNPS incidence in children is even lower than the incidence in adults (an older age was suggested as being a risk factor) (3, 5). In the pediatric cases that have been reported, the manifestation of DNPS varied, but, as in our case, children suffered from visual disturbances (21, 22). The typical basal ganglia lesions reported on MRI of adult patients with DNPS were almost absent in the pediatric case reports (as with our patient), but a cortical involvement was reported in all (3, 21, 22). We chose to present this case to raise the awareness of DNPS and the need for a close neurological follow-up of patients that underwent CO intoxication. Collecting data on these patients will help understand the risk factors, pathogenesis, and constructing treatment guidelines for this condition in children.

## Data Availability Statement

The original contributions presented in the study are included in the article/supplementary material, further inquiries can be directed to the corresponding author.

## Author Contributions

HG and GH were the doctors to diagnose the patient and conceptualized and drafted the initial manuscript. IN was the treating pediatric neurologist treating the patient and reviewed and revised the manuscript. BT was the pediatric radiologist treating the patient and reviewed and revised the manuscript. EH was the senior doctor treating the patient and reviewed and revised the manuscript. All authors approved the final manuscript as submitted and agreed to be accountable for all aspects of the work.

## Conflict of Interest

The authors declare that the research was conducted in the absence of any commercial or financial relationships that could be construed as a potential conflict of interest.

## Publisher’s Note

All claims expressed in this article are solely those of the authors and do not necessarily represent those of their affiliated organizations, or those of the publisher, the editors and the reviewers. Any product that may be evaluated in this article, or claim that may be made by its manufacturer, is not guaranteed or endorsed by the publisher.

## Abbreviations

DNPS: delayed neuropsychiatric sequel
DE: delayed encephalopathy
DNS: delayed neurological sequel
CO: carbon monoxide
COHb: carboxyhemoglobin
EF: ejection fraction
PER: pediatric emergency room
EEG: electroencephalogram
CRP: C-reactive protein
CT: computed tomography
MRI: brain magnetic resonance imaging
HBO: hyperbaric oxygen.
